# Association of plasma endotoxin, inflammatory cytokines and risk of colorectal adenomas

**DOI:** 10.1186/1471-2407-13-91

**Published:** 2013-02-26

**Authors:** Melissa Kang, Patrick Edmundson, Felix Araujo-Perez, Amber N McCoy, Joseph Galanko, Temitope O Keku

**Affiliations:** 1 Center for Gastrointestinal Biology and Disease, University of North Carolina, 103 Mason Farm Road, 7340 Medical Biomolecular Research Building, CB # 7032, 27599-7032, Chapel Hill, NC, USA

**Keywords:** Endotoxin, Inflammatory cytokines, Colonic neoplasm, Adenoma, Limulus amebocyte lysate

## Abstract

**Background:**

Recent studies suggest that bacterial endotoxins may be associated with various chronic diseases, including colorectal adenomas and cancer. Given the evidence linking inflammation and colorectal cancer, we sought to determine if plasma endotoxin concentrations are associated with indicators of systemic or local inflammation and colorectal adenomas.

**Methods:**

This cross-sectional study consisted of participants who underwent screening colonoscopies and included adenoma cases (n=138) and non-adenoma controls (n=324). Plasma concentrations of endotoxin were measured with Limulus Amebocyte Lysate (LAL) assay. We quantified concentrations of inflammatory cytokines, interleukin-4 (IL-4), IL-6, IL-8, IL-10, IL-12, tumor necrosis factor-alpha (TNF-α), and interferon-γ (IFN-γ) in plasma by ELISA and mRNA expression levels in rectal mucosal biopsies by quantitative RT-PCR. Interleukin-17 was evaluated only in the rectal mucosa.

**Results:**

Compared to subjects with low plasma endotoxin concentrations, those with higher concentrations were more likely to have adenomas (OR 1.4, 95% CI 1.0-2.1). Among subjects with adenomas, those with villous histology were more likely to have higher endotoxin concentrations (5.4 vs. 4.1EU/mL, p=0.05) and lower plasma IFN-γ (0 vs. 1.64 pg/mL, p=0.02) compared to those with only tubular adenomas. Cases showed a trend of having higher plasma TNF-α levels than controls (p=0.06), but none of the other plasma or rectal mucosal cytokine levels differed between cases and controls. Elevated mucosal IL-12 levels were associated with having multiple adenomas (p=0.04). Higher concentrations of plasma endotoxin predicted increased plasma IL-12 levels (OR 1.5, 95% CI 1.0-2.2) and rectal mucosal IL-12 (OR 1.9, 95% CI 1.0-3.7) and IL-17 gene expression (OR 2.2, 95% CI 1.0-4.6).

**Conclusions:**

These findings suggest that interactions between elevated plasma endotoxin concentrations and inflammatory cytokines may be relevant to the development of colorectal adenomas.

## Background

Colorectal cancer (CRC) is one of the leading causes of cancer death in the world and the United States [1] with the majority of cases having no previous family history. Sporadic CRC has been linked to several risk factors including accumulation of genetic mutations, inflammatory states such as inflammatory bowel disease, and environmental factors such as smoking and obesity. More recently, CRC has been linked to changes in the gut microbiota [2-5]. While bacterial dysbiosis has been associated with CRC, the mechanism by which it promotes colon carcinogenesis has not been elucidated. As adenomas are known CRC precursors, studies to determine risk factors for adenomas could be crucial to prevention and treatment of CRC. Consequently, identifying the role of gut bacteria, microbial products, and their effect on inflammation can further aid in our understanding of CRC pathogenesis.

The human intestine is a complex and unique environment inhabited by 10^13^ bacteria that interact with the intestinal mucosa to affect gut immunity and homeostasis [6-9]. An imbalance of the bacterial population in favor of pro-oncogenic bacteria could lead to abnormal proliferation of the colonic epithelium and adenoma formation [10,11]. Although previous studies have reported an association between gram-positive bacteria, *Streptococcus* gallolyticus (formerly *S*. bovis) and colorectal neoplasia [12], we have demonstrated that an increased abundance of Proteobacteria, a decreased abundance of *Bacteroides*, and a disproportionate colonization of the gut with predominant *Escherichia* coli were associated with adenomatous states [13,14]. More recently, Kostic *et al.* observed that *Fusobacterium* were enriched in CRC, also supporting a role of gram-negative bacteria in colorectal carcinogenesis [2]. One of the proposed mechanisms by which *S*. gallolyticus promotes development of colorectal tumors is through the production of inflammatory cytokines by the release of their cell wall antigens [12]. Thus, bacterial dysbiosis that favors higher abundance of gram-negative bacteria could contribute to the formation of adenomas via increased endotoxin release and inflammation.

Endotoxin, or lipopolysaccharide (LPS), is a component of the cell wall of gram negative bacteria and is released into the host environment by the destruction of the cell wall. Studies have shown that certain bacterial types are correlated with elevated concentrations of plasma endotoxin [15,16], which can have detrimental effects. Under experimental conditions, endotoxin binds to LPS-binding protein to form a complex with CD14, which leads to activation of toll-like receptor 4 (TLR-4), initiation of innate inflammatory response, activation of macrophages and monocytes, and production of pro-inflammatory cytokines such as tumor necrosis factor-α (TNF-α), interleukin (IL)-6, and IL-23 [15-20]. An overabundance of LPS-rich bacteria in the gut may provide an environment that is conducive for chronic inflammation and increased production of pro-inflammatory cytokines and reactive oxygen species. These cytokines can activate the NF-κβ pathway, which has been implicated in cell proliferation and DNA damage leading to carcinogenesis [17-20].

The aim of our study was to determine the relationship between plasma endotoxin, systemic and local inflammation, and colorectal adenomas. We assessed the association between plasma endotoxin concentrations, plasma cytokines IL-4, IL-6, IL-8, IL-10, TNF-α, and interferon-γ (IFN-γ) levels, and local cytokine mRNA expression levels of IL-4, IL-6, IL-8, IL-10, IL-17, TNF-α, and IFN-γ in relation to adenomas.

## Methods

Study population. The study population and data collection were as previously described [13,14]. The subjects in this cross-sectional study were drawn from the Diet and Healthy Study V (DHSV), where participants underwent screening colonoscopies at the University of North Carolina at Chapel Hill Hospitals. Eligibility requirements were as follows: age ≥ 30 years; proficiency in English to provide informed written consent and participate in a phone interview; a satisfactory preparation for colonoscopy and complete examination to the cecum; outpatient; no history of familial polyposis, colitis, previous colonic resection, previous colon cancer or polyps; and had not taken antibiotics within 12 weeks prior to the colonoscopy. Information about diet and lifestyle was collected by telephone interview for each subject within 12 weeks of the colonoscopy. Anthropometric measures were obtained on the day of the colonoscopy. Rectal mucosal biopsies taken at 10–12 cm from the anal verge were obtained during colonoscopy. All participating subjects provided written informed consents and consented to blood samples. Subjects who had adenomatous polyps at colonoscopy were classified as cases (n=138) and subjects without adenomas as controls (n=324). The study was approved by the Institutional Review Board at the University of North Carolina, School of Medicine (Protocol #05-3138).

Plasma endotoxin assay. Blood samples were collected in EDTA-containing tubes, and plasma was separated by centrifugation. Plasma samples were frozen in small aliquots at −80°C to prevent repeated freezing and thawing until analysis. Plasma endotoxin concentrations were measured using a commercially available quantitative chromogenic endpoint Limulus Amebocyte Lysate (LAL) QCL-1000 kit (Lonza, Walkersville, MD). Briefly, 300 μl of plasma was diluted 1:3 with 10 mM MgCl_2_ (Lonza), then heat inactivated at 70°C for 30 min, followed by further dilutions to 1:30–1:40 and 50 μl of the diluted sample was added to a 96-well pyrogen-free culture plate. Remaining procedures were performed according to the manufacturer’s instructions. Endotoxin concentrations (EU/ml) in the samples were determined from a standard curve using pure endotoxin standards. All assays were run in duplicate. The detection range of this kit was 0.1EU/ml to 1.0EU/ml. For samples that were below the lowest standard value, endotoxin concentration was calculated as the blank (negative control) concentration value of 0.055 times the dilution factor. Two samples required much larger dilutions at a ratio of 1:99 and could not be accurately compared to the endotoxin standard curve. These two samples were removed as outliers. The intra-assay and inter-assay coefficients were 4.3% and 17.2%, respectively.

Quality control for plasma endotoxin assay. Blood samples were collected in EDTA-containing tubes to prevent coagulation. Because LAL is composed of the coagulation system of the horseshoe crab, EDTA can inhibit LAL gel formation by chelating divalent cations in the LAL formulation, thus, depleting the lysate of the cations it needs to function properly. Therefore, samples were diluted in 10 mM MgCl2 to overcome the inhibitory nature of EDTA. This method was proven to pass the positive product control test by the manufacturer. Furthermore, plasma contains protein components that could inhibit the assay. To verify the lack of product inhibition, samples were tested for inhibition according to the manufacturer’s instructions. Briefly, samples were either spiked with 0.4 EU/mL of endotoxin or unspiked. Endotoxin concentrations from both samples were determined. If the difference between spiked and unspiked samples equaled 0.4 EU/mL + 25%, the sample was considered uninhibited. We performed this on groups of samples and determined that plasma containing hemolyzed red blood cells did not pass the test. Thus, only plasma samples with clear yellow color passed the test and were included in our assay. With these measures, the components potentially interfering with the reaction were minimized.

Enzyme-linked immunosorbent assay (ELISA) of plasma cytokines. ELISA was used to quantify inflammatory cytokines in the plasma samples. Any samples with gross hemolysis or lipemia were not assayed. Plasma samples were thawed and centrifuged to remove any precipitates prior to running the assay. The plasma cytokine assay was performed using the Milliplex high sensitivity human cytokine kit for IL-4, IL-6, IL-8, IL-10, TNF-α, and IFN-γ (HSCYTO-60SK, Millipore, Billerica, MA) following the manufacturer’s recommendation. Plasma samples were mixed with fluorescently labeled, color coded microspheres in 96-well plates and incubated overnight at 4°C. The next day plates were washed, and a biotinylated detection antibody was added, followed by incubation with agitation for 1 h at room temperature. Next, Streptavidin-Phycoerythrin was added to each well, followed by incubation for 30 min and another wash step. Finally, the reaction was read immediately on the Bioplex 200 System (Biorad, Hercules CA). Fluorescent intensities for the samples were derived by fitting on a standard curve. Each assay was run in duplicate with positive controls included in each batch. The average of two measurements was used for data analysis. Minimal detection levels were 0.13 pg/mL for IL-4, 0.10 pg/mL for IL-6, 0.11 pg/mL for IL-8, 0.15 pg/mL for IL-10, 0.05 pg/mL for TNF-α, and 0.29 pg/mL for IFN-γ. The intra-assay and inter-assay coefficients of variation were 4.16% and 9.12% for IL-4, 3.51% and 4.48% for IL-6, 3.26% and 6.48% for IL-8, 3.31% and 11.84% for IL-10, 3.49% and 3.78% for TNF-α, and 4.88% and 7.79% for IFN-γ respectively.

RNA extraction, reverse transcription and quantitative real-time PCR (qRT-PCR) of rectal mucosal biopsies. Rectal biopsies were placed in RNA Later (Qiagen, Valencia, CA) immediately after collection. RNA extraction was performed within 1 week of obtaining the tissue biopsies, and isolated RNA was stored at −80°C in small aliquots to prevent repeated freezing and thawing. Extraction of RNA and qRT-PCR were previously described [21,22]. Briefly, RNA was extracted from tissue biopsies using the Qiagen RNeasy Protect Mini Kit following the manufacturer’s protocol (Qiagen, Valencia, CA). RNA purity and concentration were evaluated by Agilent Bioanalyzer (Agilent, Santa Clara, CA) as well as absorbance readings using the NanoDrop ND-1000 spectrophotometer immediately after the extraction (Thermo Scientific, Wilmington, DE). Only samples with an RNA integrity number (RIN) above 7 were used for RT-PCR assays (Agilent, Santa Clara, CA). One μg of total RNA was reverse transcribed (RT) using the Cloned AMV First-Strand cDNA Synthesis Kit (Invitrogen, Grand Island, NY). Commercially available RT-qPCR primers (SABiosciences, Valencia, CA) were obtained for the housekeeping gene, hydroxymethylbilane sythase (HMBS), and seven inflammatory cytokines that included: IL-4, IL-6, IL-8, IL-10, IL-17, TNF-α, and IFN-γ. Each reaction contained 2 μL of cDNA, 1 μM of each primer, and 5 μL of Fast-SYBR Green Master Mix (Applied Biosystems, Carlsbad, CA). Cycling conditions were: 1 cycle at 95°C for 10 min followed by 45 cycles of 95°C for 15 s, 60°C for 1 min, and 72°C for 30 s. All samples were run in duplicate for both the target and housekeeping genes. The housekeeping gene was used for normalization. Pooled RNA from control subjects was included with each batch of RT-PCR reaction and served as a reference. This pooled RNA also served as a calibration point across different batches of PCR runs. Transcript abundance was calculated by the delta-delta threshold cycle (ΔΔCt) method [23]. Additional quality control measures included initial validation of qPCR efficiency for all target genes and the housekeeping gene. Standard curves were generated using dilutions series of standards for target or housekeeping genes and the PCR efficiency was calculated using the method of Pfaffl *et al.*[24]. The amplification efficiency of the target genes and housekeeping standard were comparable.

Statistical analysis. Participant characteristics were compared using student’s *t*-test or a chi squared test. Median concentrations of plasma endotoxin and inflammatory markers were compared between controls and cases by the Mann–Whitney *U* test as this data was highly skewed and not normally distributed. As a result, we used nonparametric approaches (Mann–Whitney U tests) which do not assume normality.

Plasma endotoxin concentrations in controls were used to determine upper and lower 50% cut points. Upper and lower halves of plasma endotoxin concentrations were compared between cases and controls using unconditional logistic regression and odds ratio. Plasma cytokines, tissue cytokines, BMI, physical activity, smoking status and daily fat intake were tested as potential confounders. Each was added to a logistic regression model with case status as the response and upper or lower halves of endotoxin concentrations as the predictor. If the odds ratios (OR) for the concentrations of endotoxin changed by at least 10% after the inclusion of the new variable in the model, then that variable was considered to be a potential confounder. After all such potential confounders were identified, they were all entered into the model along with the concentrations of endotoxin, and a backwards stepwise procedure was performed with endotoxin being forced into the model. Ultimately, we identified all tissue cytokines and dietary fat as confounders and ran the final logistic regression model with adjustment for these variables.

Associations between endotoxin concentrations and adenoma size, number, grade and location were also assessed by Mann–Whitney *U* test. The relationship between each plasma or rectal mucosal cytokine and endotoxin concentrations was also evaluated by multivariate logistic regression while adjusting for the levels of other plasma or rectal mucosal cytokines. P-values less than 0.05 were considered statistically significant while P-values greater than 0.05 but less than 0.10 were considered a trend toward significance. P-values were adjusted for multiple comparisons via False Discovery Rate [25].

## Results

We evaluated concentrations of plasma endotoxin and local and systemic inflammatory cytokines from 138 cases and 324 controls. The characteristics of cases and controls are shown in Table 1. Subjects were evenly distributed between males and females and were mostly Caucasians. Compared to controls, cases were slightly older and more likely to be overweight and obese. All adenomas were discovered in the colon, and only 5 subjects had adenomas with a villous component. We analyzed the data using only tubular adenomas, but results were largely unchanged from when we analyzed tubular and villous adenomas together. Thus, we present the data for all adenomas.

**Table 1:** General characteristics of study population

**Characteristics**	**Case (n = 138)**	**Control (n = 342)**	**p**
Age (years), mean (se)	57.0 (0.6)	55.2 (0.4)	0.01
Sex, n (%)			
Male	72 (55)	142 (47)	0.12
Race, n (%)			
White	121 (88)	296 (89)	0.73
Black	16 (12)	35 (11)	
BMI, n (%)			0.03
Normal	43 (32)	147 (44)	
Overweight	61 (45)	113 (34)	
Obese	32 (24)	74 (22)	
Waist/Hip ratio (mean (se))	0.934 (0.007)	0.907 (0.004)	0.001
Calories (kcal/day), mean (se)	1986 (65)	1993 (47)	0.92
Smoking Status, n (%)			0.62
Current	11 (8)	26 (9)	
Former	41 (32)	109 (36)	
Never	78 (60)	166 (55)	
Physical activity	2263 (215)	2565 (159)	0.28
(MET minutes per day), mean (se)			
Fat intake (grams/day), mean (se)	75.8 (2.8)	77.0 (1.9)	0.71
Number of adenomas, mean (se)	1.8 (0.1)		
Size of adenomas (mm), mean (se)	5.7 (0.4)		
New adenoma histology			
Tubular, n (%)	100 (95)		
Villous, n (%)	5 (5)		

The median levels of endotoxin concentrations for cases was 4.1 (1.8-26.8 EU/mL) and for controls 3.8 (1.8-26.4 EU/mL). Plasma endotoxin concentrations were divided into higher and lower concentrations based on the cut points in controls. Compared to subjects in low plasma endotoxin concentrations, those with higher concentrations were significantly more likely to have adenomas when adjusted for rectal mucosal cytokines and dietary fat (OR=1.4, CI 95% 1.0–2.1) (Table 2).

**Table 2:** Association between plasma endotoxin concentrations and presence of adenomas

**Plasma Endotoxin**	**Unadjusted OR (95% CI)**	Adjusted OR (95% CI)*
Lower Half (0–3.76 EU/mL)	1.0 (Ref)	1.0 (Ref)
Upper Half (3.80-26.39 EU/mL)	1.4 (1.0, 2.1)	1.4 (1.0, 2.1)

*OR* odds ratio, *CI* confidence interval.

*adjusted for rectal mucosal cytokines and daily dietary fat.

We also assessed whether concentrations of plasma endotoxin were associated with adenoma characteristics. The average number of adenomas was 1.8 per person (range 1–9). The adenoma size distribution was as follows: small adenoma, 1–5 mm, 66%; medium adenoma, 6–10 mm, 25%; large adenoma, >10 mm, 9% (Table 3). Endotoxin concentration was significantly higher in those with villous adenomas than tubular adenomas (p<0.05) (Table 3). However, there were no associations between adenoma size or number and endotoxin concentrations.

**Table 3:** Endotoxin concentration analysis by adenoma characteristics

	**n**	**Median (25th, 75th)**	**p**
Adenoma size			
1-5 mm	90	4.11 (2.21, 5.21)	0.44
6-10 mm	34	4.06 (2.81, 5.38)	
>10 mm	13	4.95 (3.44, 5.61)	
Adenoma number			
1-2	109	4.13 (2.54, 5.27)	0.60
3+	29	4.22 (2.08, 5.69)	
Adenoma grade			
Tubular	100	4.11 (2.19, 5.30)	0.05
Villous*	5	5.41 (4.86, 5.61)	
Adenoma location			
Proximal	50	4.14 (2.84, 5.35)	0.90
Distal	88	4.13 (2.19, 5.36)	

*Any adenomas containing villous components were classified as villous.

Median plasma or rectal mucosal cytokine levels were not different between cases and controls, with the exception of plasma TNF-α levels which showed a trend toward being higher in those with adenomas than those without adenomas (p=0.06) (Table 4, Table 5). We compared plasma and rectal mucosal cytokines between cases with one versus multiple adenomas, cases with at least one adenoma of at least 1 cm in size versus all adenomas less than 1 cm in size, and cases with at least one adenoma with villous component versus all adenomas having only tubular components, using Mann–Whitney *U* test. In subjects with multiple adenomas as compared to those with only one adenoma, only median mucosal IL-12 level was significantly higher (1.09 vs. 0.65, p=0.04). In subjects with villous adenomas, only median plasma IFN-γ was significantly lower than in those with only tubular adenomas (0 vs. 1.64 pg/mL, p=0.02). None of the other comparisons for number of adenomas or adenoma type and plasma or rectal mucosal cytokines were statistically significant (results not shown).

**Table 4:** Comparison of plasma concentration of endotoxin and plasma cytokine levels among cases and controls

**Plasma levels, units**	**Case (n = 138)**	**Control (n = 342)**	**p**	**Adjusted**
	**(median (min, max))**	**(median (min, max))**		p*
TNF-α (pg/mL)	4.8 (1.3, 276.6)	4.3 (0, 268.3)	0.008	0.06
IL-4 (pg/mL)	4.3 (0, 916.5)	2.6 (0, 5459.2)	0.07	0.19
IL-6 (pg/mL)	4.1 (0, 1099.1)	4.0 (0, 911.1)	0.70	0.80
IL-8 (pg/mL)	3.0 (0, 131.2)	2.7 (0, 291.1)	0.07	0.19
IL-10 (pg/mL)	8.5 (0, 1644.7)	7.4 (0, 1308.8)	0.60	0.80
IL-12 (pg/mL)	0.5 (0, 716.1)	0.5 (0, 1106.3)	0.82	0.82
IFN-γ (pg/mL)	1.4 (0, 924.8)	1.5 (0, 390.0)	0.57	0.80

*TNF* tumor necrosis factor, *IL* interleukin, *IFN* Interferon.

* adjusted for multiple comparisons via False Discovery Rate.

**Table 5:** Comparison of tissue cytokine levels among cases and controls

Tissue levels, *	**Case (n = 111)**	**Control (n = 278)**	**p**	**Adjusted**
	**(median (min, max))**	**(median (min, max))**		p**
TNF-α	0.8 (0.03, 18.6)	0.8 (0, 18.2)	0.92	0.92
IL-4	0.5 (0, 16.0)	0.4 (0, 14.4)	0.08	0.29
IL-6	1.0 (0.06, 41.2)	1.1 (0.1, 39.1)	0.77	0.88
IL-8	0.5 (0, 24.8)	0.5 (0, 32.4)	0.44	0.59
IL-10	0.4 (0, 115.1)	0.6 (0, 72.9)	0.05	0.29
IL-12	0.7 (0.04, 24.3)	0.8 (0.01, 16.3)	0.34	0.54
IL-17	0.7 (0, 12.4)	0.9 (0, 15.1)	0.11	0.29
IFN-γ	0.8 (0.06, 56.6)	0.7 (0.02, 24.8)	0.34	0.54

*TNF* tumor necrosis factor, *IL* interleukin, *IFN* Interferon.

*Relative gene expression to HMBS and normalized to pooled RNA of controls.

** adjusted for multiple comparisons via False Discovery Rate.

In addition, we examined the relationship between plasma endotoxin concentrations and a panel of plasma or rectal mucosal cytokines using multivariate logistic regression. We found that those in the upper half of endotoxin concentrations were significantly more likely to have higher plasma IL-12 as compared to those in the lower half of endotoxin concentrations (OR=1.5, CI 95% 1.0-2.2) (Additional file 1: Table S1). Similarly, those in the upper half of endotoxin concentrations were significantly more likely to have higher mucosal IL-12 (OR=1.9, CI 95% 1.0–3.7) and mucosal IL-17 (OR=2.2, CI 95% 1.0–4.6) than those in the lower half of the endotoxin concentrations (Additional file 1: Table S1). No statistically significant results were found for the remaining cytokines.

## Discussion

This study evaluated the relationship between endotoxemia and colorectal adenomas and their association with systemic and mucosal cytokines. We found an association between high endotoxin concentrations and adenomas. Compared to those with low endotoxin concentrations, those with higher endotoxin concentrations were more likely to be cases. We also observed a positive association between villous adenomas and high endotoxin concentrations. Only plasma TNF-α levels showed a trend toward being higher in adenoma cases than controls (p=0.06). None of the other plasma or rectal mucosal cytokine levels was significantly different between cases and controls. In multivariate analysis, higher concentrations of endotoxin predicted increased plasma levels of IL-12 as well as rectal mucosal gene expression levels of IL-12 and mucosal IL-17. Increased mucosal gene expression level of IL-12 was also associated with multiple adenomas.

We found that subjects with adenomas were more likely to have higher endotoxin concentrations, especially those with villous adenomas. High dietary fat intake has been shown to increase endotoxemia [26], and in our study, positive relationship between adenoma status and endotoxemia was maintained even after adjusting for rectal mucosal cytokines and dietary fat. Limited studies have evaluated the association between endotoxins and colorectal adenomas. A recent report by Lee *et al.* demonstrated that endotoxin concentrations were higher in individuals with polyps, especially dysplastic adenomas. Our results are consistent with their findings [27]. To our knowledge, these are the first studies to show that higher endotoxin concentrations are associated with adenoma risk in humans. While Lee *et al.* did not evaluate any mechanisms that could explain the endotoxin relationship to adenomas, we propose that bacterial dysbiosis in favor of over-abundance of gram-negative bacteria in individuals with adenomas could lead to increased endotoxin release, and thereby contribute to elevated production of inflammatory cytokines. This, in turn, could promote leakiness of the intestinal mucosal barrier and translocation of endotoxin into the blood stream. A recent study showed that intraluminal administration of LPS in the colon resulted in altered local cytokine production suggesting that elevated LPS in the colon is able to cause intestinal inflammation [28].

Chronic inflammation is a risk factor for colorectal cancer. We have previously shown that elevated levels of plasma IL-6 and TNF-α are associated with increased risk of adenomas [21]. In this study, we found a borderline association between plasma TNF-α levels and colorectal adenomas. Evaluation of the relationship between endotoxin and plasma or mucosal cytokines revealed that plasma IL-12 levels, and rectal mucosal levels of IL-12 and IL-17 were likely to be elevated with increased endotoxin concentrations. This observation for IL-12 is consistent with published literature demonstrating imbalance in Th1 cytokine network in colorectal carcinogenesis [29,30]. IL-17 is a pro-inflammatory cytokine that could promote tumorigenesis and tumor progression [31,32]. Our observations support an association between plasma endotoxin concentrations and markers of systemic and mucosal inflammation.

When we analyzed rectal mucosal and plasma cytokines in relation to adenoma characteristics, we found that those with villous adenomas were more likely to have lower plasma IFN-γ. IFN-γ is mainly induced by IL-12. Elevated levels of IL-12 have been reported in adenomas while decreased levels have been noted in CRC [29,30]. As villous adenomas are more likely to be dysplastic than tubular adenomas and closer in progression to CRC, it is possible that changes in cellular immunity such as altered levels of immune markers, IL-12 and IFN-γ, could contribute to cancer development. Our results demonstrate that rectal mucosal inflammatory cytokines are positively associated with plasma endotoxin concentrations, particularly for adenoma cases. These results are consistent with prior literature showing a correlation between endotoxemia and inflammatory cytokines and oxidative stress markers, and suggest a role of microbiota in mediation of inflammation and adenoma formation via endotoxin production [14,33].

Given that the gut is colonized by complex bacterial communities, we acknowledge that elevated endotoxin concentrations alone may be insufficient to promote inflammation and adenomas. It is likely that other bacteria or bacterial products may be involved. For, example, *Streptococcus* gallolyticus (formerly *S*. bovis), a gram-positive bacteria, has been associated with colorectal adenomas and cancer [12]. *S*. gallolyticus is thought to promote increased production of inflammatory cytokines and development of colorectal tumors via the release of cell wall antigens [34]. However, our previous studies, demonstrating an increased abundance of LPS-rich Proteobacteria, particularly *Escherichia* coli, in association with adenomatous states [13,14] provide a good rationale for evaluating bacterial endotoxins, inflammation and adenomas.

In this study, we used mucosal biopsies obtained from the rectum while the adenomas were found in the colon. The rectum has been routinely sampled in human studies to assess biomarkers of colorectal adenomas and cancer. This is based on the idea of a “field effect” whereby events occurring in the rectum are reflective of events going on elsewhere in the colon [35-39]. Ponz de Leon *et al.* examined mucosal proliferation in different tracts of the large bowel in subjects with colorectal polyps or cancer and normal controls [40]. They found no significant differences in cell proliferation between mucosal samples taken at various distances from the colorectal cancer margin suggesting that hyper-proliferation of the entire colonic mucosa was common in patients with colorectal cancer. Furthermore, we have previously established that decreased apoptosis is a risk factor for adenomas, and lower rates of apoptosis can be detected from normal rectal mucosa distant from adenomatous tissue [36]. This field effect has also been observed with bacterial composition. Momozawa *et al.* examined seven sites in the gut and noted that there were no significant quantitative or qualitative differences in bacteria from ileum to rectum, suggesting that the majority of bacteria would be similar throughout the large intestine [41]. These studies support that rectal mucosal biopsies could be a good surrogate for biopsies adjacent to adenomas.

We did not have comprehensive medical histories of the subjects. While endotoxin transfer to the bloodstream could be caused by other factors such as tissue damage, infection and other medical conditions that may induce systemic inflammation, all the subjects in our study were ambulatory and healthy enough to undergo outpatient screening colonoscopies. Although, we did not have detailed past infection histories, one of the inclusion criteria was that subjects had not taken antibiotics within 12 weeks prior to colonoscopy, thus, ruling out recent bacterial infections contributing to inflammation.

A limitation of our study is that we used a commercially available endpoint chromogenic LAL test with detection level as its low as 0.1EU/ml. We chose this method because of its ability to quantitate plasma endotoxin as well as low overall equipment cost. Even though we controlled for protein inhibition of the LAL endotoxin assay, it is possible that there were still components that could interfere with the reaction and potentially obscure the distinction between cases and controls. However, our results are comparable with other studies that evaluated presence of plasma endotoxin in healthy individuals [42-44]. Lastly, we did not measure endotoxin concentrations in the mucosa, which could be more relevant to adenoma formation. We also recognize that with the cross-sectional nature of our study, we cannot establish a causal relationship between endotoxin, inflammation and colorectal adenomas, and that studies in animal models are needed to assess potential mechanisms. This study has several strengths which include a large sample size, detailed exposure information, and evaluation of both local and systemic markers of inflammation.

## Conclusion

To the best of our knowledge, this is one of the first studies to evaluate the association of plasma endotoxin and inflammation in relation to the risk of colorectal adenomas. We found a positive association between plasma endotoxin concentrations and adenomas as well as several measures of rectal mucosal inflammation. In particular, higher levels of endotoxin predicted increased plasma and mucosal IL-12 and mucosal IL-17. Those with villous adenoma histology were more likely to have higher endotoxin but lower plasma IFN-γ as compared to those with tubular histology. Thus, our study suggests that bacterial endotoxins are associated with increased levels of plasma and rectal mucosal cytokines, especially in subjects with adenomas.

## Abbreviations

CRC: Colorectal cancer;IFN: Interferon;IL: Interleukin;LPS: Lipopolysaccharide;TNF: Tumor necrosis factor

## Competing interests

The authors declare that they have no competing interests.

## Authors’ contributions

MK: planned the details of the study, collected and interpreted data, and drafted the manuscript. PE: collected data. ANM: collected data. FAP: collected data. JAG: performed statistical analysis. TOK: planned the study, interpreted data, and drafted the manuscript. All authors read and approved the final manuscript.

## Pre-publication history

The pre-publication history for this paper can be accessed here:

http://www.biomedcentral.com/1471-2407/13/91/prepub

## Supplementary Material

Additional file 1: Table S1. Odds ratios (95% CI) for elevated levels of cytokines given elevated endotoxin concentrations. Click here for file
