# Supplementary material for: Association of plasma endotoxin, inflammatory cytokines and risk of colorectal adenomas
Source: BMC Cancer. 2013 Feb 26;13:91. doi: 10.1186/1471-2407-13-91 (PMC3599094; doi:10.1186/1471-2407-13-91)
Supplement: Additional file 1: Table S1. — Odds ratios (95% CI) for elevated levels of cytokines given elevated endotoxin concentrations. [file 1471-2407-13-91-S1.docx]

Supplementary Table 1. Odds ratios (95% CI) for elevated levels of cytokines given elevated endotoxin concentrations.

| Cytokines | OR (CI 95%) |
| --- | --- |
| Plasma TNF-α | 1.2 (0.8-1.7) |
| Plasma IL-4 | 0.7 (0.5-1.0) |
| Plasma IL-6 | 0.8 (0.5-1.1) |
| Plasma IL-8 | 0.7 (0.5-1.1) |
| Plasma IL-10 | 0.8 (0.6-1.3) |
| Plasma IL-12 | 1.5 (1.0-2.2) |
| Plasma IFN-γ | 1.0 (0.7-1.5) |
|  |  |
| Tissue TNF-α | 0.9 (0.5-1.5) |
| Tissue IL-4 | 1.1 (0.7-1.8) |
| Tissue IL-6 | 1.4 (0.8-2.4) |
| Tissue IL-8 | 1.2 (0.7-2.0) |
| Tissue IL-10 | 0.8 (0.5-1.4) |
| Tissue IL-12 | 1.9 (1.0-3.7) |
| Tissue IL-17 | 2.2 (1.0-4.6) |
| Tissue IFN-γ | 1.1 (0.5-2.3) |
